# Supplementary material for: ClarID: A Human-Readable and Compact Identifier Specification for Biomedical Metadata Integration
Source: medRxiv. 2025 Sep 7:2025.09.05.25335150. Preprint. [Version 1] doi: 10.1101/2025.09.05.25335150 (PMC12424912; doi:10.1101/2025.09.05.25335150)
Supplement: Supplement 2 [file media-2.pdf]

Supporting Table 1: Implementation challenges and solutions

**Data pre-processing**	
**Faced challenges**	**Solution**
CSV is flexible but unstructured; missing values and inconsistent codes are common	We developed a preprocessing script, guided by a configuration file, to standardize raw CSV rows and columns. The script handles missing values, harmonizes inconsistent codes, and produces a clean CSV suitable for bulk processing with ClarID-Tools. The scripts are available at: https://github.com/CNAG-Biomedical-Informatics/clarid-tools/tree/main/nb/data/scripts
**CLI + Module**	
**Faced challenges**	**Solution**
Standardization of components' vocabulary	In the codebook, ontology terms were used to define properties where possible (e.g., tissues with UBERON). For diseases and technical fields like batch/replicate, regex rules were applied. The structure remains flexible, allowing users to extend it as needed.
Externalized schema vs. hard-coded specification	Full JSON Schema externalization proved impractical; we adopted a hybrid approach with YAML-configurable parameters and some hardcoded elements, while validating the codebook itself against a JSON Schema.
Compaction of human → stub formats	Certain fields required Base62 encoding to condense information. The field width was restricted for parsing but can be adjusted through CLI parameters.
