# Supplementary material for: ClarID: A Human-Readable and Compact Identifier Specification for Biomedical Metadata Integration
Source: medRxiv. 2025 Sep 7:2025.09.05.25335150. Preprint. [Version 1] doi: 10.1101/2025.09.05.25335150 (PMC12424912; doi:10.1101/2025.09.05.25335150)
Supplement: Supplement 3 [file media-3.pdf]

Supporting Figure 1

(a)

AsthmaCohort-01002-Control-J98.51-Female-A50_59

AsthmaCohort-01004-Case-J98.51-Male-A40_49

COPDStudy-01001-Case-J98.51-Male-A40_49

COPDStudy-01003-Control-J98.51-Female-A50_59

(b)

CNAG_Test-DanRer-00003-BLO-NOR-WES-I46-SUR-P1M-B03-R01

CNAG_Test-HomSap-00001-LIV-NOR-RNA-C22.0-BSL-P0D-B01-R05

CNAG_Test-MusMus-00002-BRN-TUM-CHI-C71.0-TRT-P7W-B02-R02

CNAG_Test-RatNor-00004-KID-NOR-LCMS-C66-CHL-P3Y-B01-R10

Figure SF1. Example QR (quick-response) codes with their decoded ClarID strings shown above each image, for (a) a "subject" entity and (b) a "biosample" entity. The codes can be read by any QR-enabled device, such as a smartphone.
